# Coccyodynia—Syrup of Licorice Root

**Published:** 1874-07

**Authors:** John O’Riley


*THE AMERICAN PRACTITIONER*—*June*, 1874:

Coccyodynia—*By John O'Riley, M.D.*—* * * One point I wish to show from the clinical history of this case is that an assemblage of symptoms which authors attribute solely to irritable and other conditions of the uterus do not depend exclusively on such morbid states, though they may co-exist with them. My reason for this is that the limiting of these symptoms to pathological conditions of the uterus prevents the practitioner from seeking the cause elsewhere, and leads to much of our unsuccessful medication. * * *

The removal of the coccyx, not only relieved the coccyodynia, but also the symptoms of pelvic muscular neuralgia mentioned by Hodge as having their origin in uterine trouble. This operation shows that these sensations, though co-existent with a diseased uterus, did not depend on it. In this case they were relieved by the removal of a diseased bone, although the womb was still considerably enlarged and suffering from chronic endometritis. If morbid effects are only relieved by eradicating the cause, then in this instance the cause was the coccyx and not the uterus. Such being the case, should we not infer that the group of symptoms daily complained of by females is often caused by some morbid condition existing in the pelvic, muscular and nervous structures themselves, and not in the womb. * * *

Such being the case, had not this assemblage of symptoms better be classed under the head of "pelvic myalgia" instead of morbid conditions of the uterus? Arranged under such a heading we will, when they are complained of, examine all the structures of the pelvis before locating their origin. We once knew a patient who, complaining of these symptoms, was treated by a prominent practitioner for months for uterine disease, when, passing into other hands, she was relieved entirely by being cured of a strictured rectum. Had this patient been thoroughly examined the proper cause would have been discovered at first. So in other cases the location will be found in the different pelvic structures, and frequently, as in Mrs. M., in the coccyx or its attachments.

